# Experiences of contacts with diabetes care professionals among people with type 2 diabetes in an urban Swedish setting

**DOI:** 10.1080/02813432.2025.2572514

**Published:** 2025-10-14

**Authors:** J. Thylefors, M. Annersten Gershater, E. Mangrio, S. Zdravkovic

**Affiliations:** Faculty of Health and Society, Malmö University, Malmö, Sweden

**Keywords:** Diabetes care, patient experiences, patient satisfaction, primary healthcare, type 2 diabetes

## Abstract

**Background:**

High-quality diabetes care should offer personalized treatment and support self-management to reduce complications and maintain quality of life for affected persons. In Malmö, Sweden’s third-largest city, primary care treated twice as many individuals with type 2 diabetes (T2D) in 2018 compared to 2011. As the prevalence of T2D increases, healthcare services face increasing challenges.

**Objective:**

To explore experiences of contacts with diabetes care professionals among people living with T2D who are accessing diabetes care in the city of Malmö.

**Method:**

A qualitative inductive design was employed, involving interviews with 21 persons with T2D receiving diabetes care at four primary healthcare centers in Malmö. A semi-structured interview guide was used. Data were analyzed using qualitative content analysis.

**Result:**

The theme ‘Seeking room for one’s agency’ emerged from two categories that illustrate experiences with diabetes care in relation to meeting healthcare needs: ‘Importance of connecting with diabetes care professionals’ and ‘Concerns in navigating diabetes care.’ Eight subcategories describe what contact with diabetes care meant for the participants.

**Conclusion:**

The findings highlight opportunities for improving diabetes care to better meet patients’ needs. Contacts with diabetes care professionals were perceived as supportive when there was a clear intention to adapt to individual needs and when expectations and communication were transparent. Continuity with general practitioners and educational aspects were identified as unmet healthcare needs. Based on the findings of this study, efforts to a systematic and broad implementation of group-based T2D-education in Malmö are recommended.

## Background

Cities are home to over half of the global population, and this urban proportion is expected to rise, with two out of three people projected to live in cities by 2050 [1]. Urban living can significantly impact health negatively, contributing to conditions like asthma and allergies, and—though with a more variable association—obesity and type 2 diabetes (T2D). However, it also offers advantages, such as better geographic access to healthcare [2].

T2D, the focus of this study, is increasing worldwide [3]. This trend is also evident in Malmö, Sweden’s third-largest and one of its most segregated cities [4]. In 1964, the diabetes prevalence in Malmö was 1.5% [5], but by 2018, the prevalence of T2D alone had risen to 4.3%. Furthermore, T2D has become more prevalent among young adults in Malmö. Between 2011 and 2018, the number of persons aged 0–29 with T2D increased from 32 to 148, and those aged 30–39 increased from 70 to 325 [6]. Additionally, the prevalence varies across residential areas within the city, ranging from 2.6% to 6.4%. Areas with lower socioeconomic status have a higher prevalence compared to areas with higher socioeconomic status [7].

The general rise in T2D, despite varying prevalence across subgroups, is considered multifactorial and can be explained at various levels [8]. In Malmö, a large proportion of the population has ethnic backgrounds that are genetically more prone to developing T2D compared to individuals of Swedish origin [8,9]. There is also an increase in risk factors for developing T2D, such as rising obesity rates [10] and a more sedentary lifestyle is common in Western societies [11]. These factors likely contribute to the rise in T2D in Malmö.

Although T2D is typically a self-managed disease [12], the affected persons rely on healthcare services for education and support to manage it effectively, reduce risks of complications, and maintain quality of life [13]. In Sweden, primary healthcare is responsible for providing medical assessment and treatment, nursing care, preventive work, and rehabilitation that do not require specialized medical or technical resources [14]. This means that persons with T2D are mainly cared for T2D within primary healthcare across the country. Healthcare and medications are primarily funded through taxes, with patients covering a minor portion of the costs through fees [14,15]. People living with T2D in Sweden commonly see a general practitioner (GP) and a diabetes nurse (DN) in primary healthcare for their diabetes management. Ideally, dietitians, physiotherapists, foot care professionals, and healthcare social workers are part of the diabetes team and consulted as needed. In complicated cases or when complications arise, check-ups and treatment can be provided at hospital-based clinics. Similarly, specialized care is involved in routine diabetes management for retinal examinations [16]. Diabetes care in Sweden thus consists of a network of different care levels and professionals.

Various aspects of access and continuity, particularly relational continuity with diabetes care professionals, have been identified as important healthcare needs among patients with T2D in Sweden and elsewhere [17–20]. Previous studies on experiences of diabetes care have also shown that people living with diabetes have a need to learn more about how to manage diabetes in practice, and not just in theory [17,18]. Sufficient time during routine encounters in diabetes care has also been stressed as important for perceived high-quality diabetes care [20]. Moreover, patient satisfaction with diabetes care has been associated with care that is characterized by being patient-centred [21].

Patient education is a cornerstone in diabetes care [13,16]. In addition to the individual consultations in routine diabetes care, various formats have been implemented or tested, such as group-based [22] and digital interventions [23], including culturally adapted approaches [24,25], to meet preferences, needs, and circumstances related to mobility or geographic location.

There have been no major changes in the organization of diabetes care in Sweden over the past decades. However, new treatments and technologies have emerged and become widely used [26]. These new and more effective therapies, along with the increasing number of persons affected by T2D, are introducing new dimensions to diabetes care. An example of the increasing demands is that the primary healthcare system in Malmö had to treat twice as many individuals with T2D in 2018 as it did in 2011 [6]. This trend is also observed elsewhere in Europe, leading to reorganizations and up-scaling of diabetes care [27,28].

Given that the number of patients requiring treatment for T2D in Malmö has recently doubled, and that access to and sufficient time during routine encounters are key factors in patients’ perception of high-quality care, it is increasingly relevant to explore how persons living with T2D in Malmö experience their contact with diabetes care. Therefore, the aim of this study was to explore experiences of contacts with diabetes care professionals among people living with T2D who are accessing diabetes care in the city of Malmö.

## Methods

A qualitative inductive design was chosen for this study, allowing room for new insights and suggestions that are not predetermined. This method is effective for gaining a broad and deep understanding of individual lived experiences [29]. The study was conducted in accordance with the Consolidated Criteria for Reporting Qualitative Research (COREQ) [30] to ensure transparency in the process.

### Setting and participants

The study was conducted in Malmö, a city in southern Sweden with 365,644 inhabitants [31]. The inclusion criteria were persons diagnosed with T2D for at least six months, aged over 18 years of age, and receiving diabetes care in Malmö. Participants were recruited through convenience sampling (see Table 1) from four primary healthcare centers, purposefully selected for their location in socio-demographically diverse areas of the city. Two of the primary healthcare centers were located in the eastern parts of the city with residents of lower socioeconomic status than average, and two of the primary healthcare centers were located in the western parts with residents of high socioeconomic status. Subsequently, DNs (*n* = 5) from these four primary healthcare centers facilitated the recruitment of persons who met the inclusion criteria.

**Table 1.** Participants characteristics.

Participants (n)	21
Median years of age (min, max)	64 (35–81)
Sex (female/male)	9/12
Family situation	
Living alone	5
Married or living with a partner	14
Living with a parent	2
Educational background	
Primary school	5
Upper secondary education	6
Higher education	10
Occupation	
Unemployed	2
Retired	13
Employed	5
Day activity for people living with disabilities	1
Born in Sweden	12
Born in another country in Europe	2
Born in a non-European country	7
Median years since diagnosed with T2D (min, max)	10 (1–30)
Insulin-treated (n)	6

The DNs were instructed to ask 5–10 patients during their check-up visits if they were interested in participating in an interview study about their experiences of living with T2D and diabetes care. Patients expressing interest were provided with written information about the study during the visit. If they consented, the first author later called to provide further information and answer any question about the study and potential participation. DNs from the primary healthcare centers in the eastern part of the city (*n* = 3) facilitated contact with 13 potential participants and DNs from the primary healthcare centers in the western parts of the city (*n* = 2) facilitated contact with 12 potential participants. A total of 25 patients consented to be contacted for further information about the study; one was not reachable, and three had changed their minds about participating. If the person consented to participate, a date and location for the interview was arranged. Interviews were conducted at Malmö University (*n* = 16), in the participant’s home (*n* = 4), or at the participant’s workplace (*n* = 1). None of the participants were acquainted with the interviewer prior to the interviews.

### Data collection

Data were collected through individual interviews using a semi-structured interview guide. This approach allowed the participants to explore angles they deemed important [29]. The interview guide, specifically designed for the study (see Table 2), had a broader range of themes concerning experiences of living with T2D in Malmö today and consisted of several themes. For this study, the data concerning the theme ‘experiences of contact with diabetes care’ were used and the other themes were excluded.

**Table 2.** Semi-structured interview guide.

1. How are you generally?
2. What are your expectations for a visit to diabetes care services?
3. How do you usually feel after a visit to diabetes care service?
4. Can you provide examples of something that works well when you have contact with diabetes care services?
5. Do you see anything that could be improved in your contact with diabetes care services?
6. If you were treated for diabetes in your home country or elsewhere in Sweden, what was your contact with healthcare like there?
7. How do you feel about the advice and support you receive from diabetes care services regarding lifestyle habits, such as diet and physical activity?
8. Is there anything you want to share that we have not talked about and that you think is important in this context?

Open-ended questions were asked about participant’s experiences with diabetes care, aiming to reflect both met and unmet healthcare needs. The interview guide’s questions were asked in a flexible order based on the participant’s responses, thus supporting the context of the experiences the participant wished to share. Probing questions, such as ‘Can you give an example?’, and clarifying questions by rephrasing an answer, such as, ‘You then mean that…?’, or ‘Is it correct that you feel that…?’, were posed depending on the participant’s narrative, in a way aiming to not to influence or distort [29]. Sparse field notes were taken to document specific circumstances that arose during the interview.

In one interview, a spouse participated alongside the participant, in accordance with the participant’s wish. In two of the interviews conducted at the participants’ home, family members were present in adjacent rooms. Two of the interviews were conducted with the help of certified interpreters in Arabic and Swedish, one of whom attended in person while the other interpreted *via* telephone. The interviews took place from August 2024 to January 2025 and were conducted by the first author. Participants were informed that the first author is a registered nurse and PhD student, and that the study is part of a thesis on different aspects of T2D in Malmö. Before recording, participants’ background data were collected and documented in writing, as presented in Table 1. Immediately thereafter, the recording began, and interviews were conducted. The interviews lasted from approximately 30 to 90 min (median = 47 min), and covered four themes, one of which was experiences of contact with diabetes care professionals, which is used in this study. The interviews were transcribed verbatim shortly after completion. No pilot interviews were conducted, and all completed interviews are included in the analysis.

### Data analysis

The transcripts were analyzed using qualitative content analysis to identify patterns and variations in the collected data [32–34]. After all interviews were conducted, the transcripts were read repeatedly by the first author to gain a comprehensive understanding of the narrated experiences.

The full semi-structured interview guide covered a broader range of themes concerning experiences of living with T2D in Malmö today. For the current study we narrowed down the analysis to data concerning experiences of contacts with diabetes care as the empirical material encompassed rich data on this specific topic.

Sentences and paragraphs, i.e. descriptive content, interpreted as meaning units relevant to the current study’s aim were then condensed and coded [32]. Thereafter, a re-contextualizing step was undertaken by combining the separated codes into new patterns based on their similarities and differences. Codes were then checked against their contexts to verify that they maintained consistency to its origin. This back-and-forth movement was continuously undertaken throughout the process to allow for a deeper understanding of the phenomena in question and to make sense of both the words used and the person who said them [34]. Through an interpretative process, clusters of codes with manifest content were then abstracted to a more latent level, while still remaining close to the concrete experiences. This step was revised several times together with the co-authors to ensure the sub-categories remained true to the participants’ experiences and to analyze the essence of the text excluded from the analysis [33]. The subcategories were then clustered in two categories, thus reflecting their latent content. Lastly, the subcategories and categories were analyzed with an increased degree of abstraction and interpretation, which formulated the theme for the rich data. An example of the process is presented in Table 3.

**Table 3.** Examples of the qualitative analysis process.

Meaning unit	Condensed meaning unit	Code	Subcategory	Category	Theme
A little bit more about food and things like that. But no, I don’t think you really get anything. You usually get a brochure—that’s how it tends to be. (11)	More information is wanted about food, but usually, only a brochure is provided	Oversimplifications in response to the need for guidance	To get through to each other (Codes: To know what applies, Meeting no true engagement, Oversimplifications in response to the need for guidance, Verbal maltreatment, Approach in accessibility is crucial)	Importance of connecting with diabetes care professionals (Subcategories: To get through to each other, A desire for a forum to share experiences and knowledge, Need for coherence in the care relationship, Personalized care contributes to feeling secure)	Seeking room for one’s agency (Categories: Importance of connecting with diabetes care professionals, Concerns in navigating diabetes care)
One time he was supposed to call me at nine, but no one called the entire morning. So, I went down to the healthcare centre, knocked on the door and walked in to see him and said: “You were supposed to call me at nine.” “Well, I haven’t had time yet.” After that, I no longer trusted that doctor. But he’s changed a bit since then. (7)	Confronting the GP when not being contacted as decided	Feedbacking when not content	Demands to take control of one’s care (Codes: Requesting a new GP, Changing healthcare center to receive competent care, Feedbacking when not content, Minimizing contact with diabetes care)	Concerns in navigating diabetes care (Subcategories: Doubts about the level of care, Need for a more flexible approach, Demands to take control over of one’s own care, Diabetes care is just a routine control)	

### Ethics approval and consent to participate

Potential participants received written information about the study before deciding whether to participate. This information was repeated orally when contacting them to invite them to take part. Written informed consent to participate was obtained from the participants, who were informed that they could withdraw from the study at any time without providing a reason. The information document and informed consent were translated into Arabic by a certified translation agency. The collected data and personal information were handled confidentially; all data were de-identified, and any identifying information was removed during transcription. The material was stored securely in locked cabinets and on password-protected computers, accessible only to the research team. The study was approved by the Swedish Ethical Review Board (reference number 2024-01046-01).

## Findings

Contact with diabetes care was a recurring activity for the participants, ranging from one or two visits per year to bi-weekly visits for sensor changes when using a continuous glucose monitor, which was performed in primary healthcare. More frequent visits were required for the treatment of diabetes-related foot ulcers, which was performed both in primary healthcare and hospital clinics. In addition to visits to GPs and DNs in primary healthcare, diabetes care also included visits to dietitians, psychologists, specialists in nephrology, diabetes-related ulcer clinic, and ophthalmic care.

### Seeking room for one’s agency

The participants described both a sense of capability and perceived opportunities to influence their contact with diabetes care professionals to meet their personal needs. This fundamental experience is reflected in the main theme: ‘Seeking room for one′s agency.’ The categories illustrate how participants relate to their contact with diabetes care professionals to have their needs met. The subcategories capture the diverse experiences of what contact with diabetes care meant to the participants, thus encompassing both met and unmet healthcare needs. A scheme of the constructed theme, categories, and sub-categories is presented in Table 4.

**Table 4.** Scheme of constructed theme, categories, and Sub-categories.

Theme	Seeking room for one’s agency							
Category	Importance of connecting with diabetes care professionals				Concerns in navigating diabetes care			
Subcategory	To get through to each other	A desire for a forum to share experiences and knowledge	Need for coherence in the care relation-ship	Personalized care contributes to feeling secure	Doubts about the level of care	Need for a more flexible approach	Demands to take control of onés care	Diabetes care is just a routine control

### Importance of connecting with diabetes care professionals

This category addresses experiences stemming from relational aspects of diabetes care. Interpersonal contact was important to all participants and was the primary experience of diabetes care for some. These experiences reflected both needs that were addressed and those perceived as subject to varying degrees of modification.

#### To get through to each other

A prominent expectation of diabetes care was that it should be accessible and include regular invitations to check-ups. Accessibility during phone hours was also emphasized as fundamental but not always experienced. Clarity from both the diabetes care providers and the participants was described as necessary. For example, one participant did not appreciate being forced to use the chat function to contact diabetes care. After making this clear to the healthcare provider, they were happily exempt from this communication method, allowing them to speak on the phone or visit in person instead. Furthermore, another participant, who suffered from complex conditions affecting well-being more than T2D, found it reasonable when the GP clarified that only diabetes-related factors would be prioritized during regular visits. This clarity provided some contentment, as the participant knew what to expect.

In contrast, communication with diabetes care was at times characterized by ambiguity, and participants experienced they received oversimplified responses. For example, when asking about food choices, they were given a pamphlet instead of engaging in a dialogue about dietary options. Being advised to rely on a food labeling symbol was perceived as insufficient for supporting healthy food decisions. Moreover, experiencing health care providers tip-toeing around the risk of complications led to uncertainty about what applied, resulting in the need for extensive self-education:

My diabetes nurse, she’s good. But she doesn’t tell me that if my sugar levels don’t go down now it will mean this and that. It’s difficult when you must read and learn everything on your own. [5]

Specific questions, such as why lowering blood glucose levels through walks did not apply to the participant’s experience, were left unanswered and with no attempt to engage in the matter. This left the participant with a feeling that there wasn’t much point in visiting the diabetes care services. One participant raised concerns about how diabetes was perceived in diabetes care, noting that physical activity had never been discussed. They expressed a desire for a more coaching approach, with positive feedback on weight loss and lifestyle changes.

Receiving the diagnosis of T2D in a non-pedagogical manner was distressing, as the GP threatened lifelong insulin treatment in the same conversation, which occurred just before the weekend, leaving no possibility to contact the caregiver. The participant had always been frightened of the idea of insulin treatment and felt terrible. Another distressing moment shared was being shouted at with a derogatory term for being obese. This incident occurred several years ago but left a lasting impact on the participant.

#### A desire for a forum to share experiences and knowledge

Participants who had lived with T2D for a long time shared positive experiences related to attending the previously existing diabetes school in Malmö. They expressed a desire for ongoing opportunities to participate – not only at the time of diagnosis, but throughout their lives. They also wished for practical components, such as cooking diabetes-friendly meals together, to be included. None of the participants described participating in any group-based training or support programs for persons with T2D. However, a significant number, mainly women, expressed a desire for a forum to learn, meet others, and share experiences, such as through a diabetes school, seminars, group meetings, or educational sessions. They considered it natural for such activities to be organized by the healthcare center:

I don’t go to group workouts or anything like that. But I could still imagine if there could be some kind of… maybe a healthcare center like the one where the diabetes nurse works. She might be able to gather a group or something, where people could talk about this [living with T2D]. And perhaps especially how diabetes is connected to everyday life, combined with vision issues. [20]

### Need for coherence in the care relationship

Several participants described having short-term relationships with their GPs, as the doctors often left or were only temporarily placed at the healthcare centre due to educational rotations. This perceived lack of personal continuity in primary care was also seen to negatively affect the continuity of information. As one participant expressed:

I know they change, but when that happens, it’s new for each of us – both that I am new to him and that he is new to me. And then he must read the medical records; he doesn’t know anything about me. I do think one should have a doctor for a longer period of time [13].

It was also noted that prescription adjustment was made by several different GPs, which created a certain sense of uncertainty. In one case, the lack of continuity in care was linked to moving to Malmö, where the person saw an ophthalmologist for a non-diabetes-related eye condition. This led to misunderstandings and the person not being invited to routine retinal screenings for several years.

When continuity was perceived in the relationship with a caregiver who also showed genuine care – by being kind, attentive, and truly seeming to care about the person – the experience of a visit to the caregiver was described as feeling like visiting a friend.

#### Personalized care contributes to feeling secure

Being monitored at regular intervals created a feeling of being seen and cared for and instilled a sense of security among the participants. This sense of security was noted both among some of them who had been living with T2D for many years and to those diagnosed in recent years.

I’m very satisfied now—I have these check-ups all the time. I feel comfortable and safe. [1]

Having come from another country and witnessed a close relative struggle with T2D, the diabetes care provided in Sweden was highly appreciated in comparison. The professionals in Sweden’s diabetes care system made adjustments based on individual needs. One participant, who had difficulties with taking blood tests, was provided with a continuous glucose monitor. This monitor worked well and contributed to a sense of confidence in managing blood glucose measurements. Furthermore, the approach was perceived as personalized, as the DN respected a participant’s preference not to be informed about measurement values. A supportive approach from the DN, or in some cases a dietitian, allowed for the exclusion of certain foods to be replaced by adjusting quantities instead. This was experienced as a relief and contributed to a sense of security in managing everyday life.

### Concerns in navigating diabetes care

This category addresses how the structure of diabetes care influences the overall experience of patient-care interactions. It highlights that participants seek to find the best path forward in their interactions with the diabetes care, aligning with their own preferences and needs.

#### Doubts about the level of care

T2D was referred to as a serious disease. Receiving specialized care for minor conditions led to reflections on whether diabetes care should be provided solely within primary healthcare. There were doubts about whether individual factors, such as age and ethnicity, were adequately considered in the assessment and treatment process. In addition, there was a desire to discuss such matters with an endocrinologist:

So, I might have met someone specialized in diabetes, that would have been interesting. Because as it is now when they diagnose, it could have been done by anyone, as they just look at the values: this is the glucose level and this is the long-term glucose level. They only see a table. And if it’s slightly too high, then it’s a diagnosis, without reflecting on who the patient is or what their background is. It’s almost like just measuring someone’s BMI; it doesn’t tell you much. It could just be muscle; or if it’s on the other side, it could be fat. I think it’s a bit too mechanical. Have they considered me as a patient? Maybe they have, but I don’t get that feeling. [19]

Experiencing multiple complications and transitioning from specialist care at a hospital clinic to primary healthcare led to a feeling of no longer receiving the most competent care. When changes were made to the treatment prescribed by primary healthcare during a hospital stay, it raised questions about the approach taken in primary care.

#### Need for a more flexible approach

In some cases, contact with diabetes care was described as being protocol-driven. This approach led to certain areas being covered too ambitiously, while others were lacking, according to the participants:

I think they go from 0 to 100. Then they follow their schedule: now we’ll prick your feet, and now we’ll do this. Instead of talking more about diet-related issues, showing the sugar levels, I don’t think they really take the time to discuss why you have this, why the values are increasing like this. Do you eat sweets? Maybe someone could have said that. Just like they sit down and check your feet, they could go over a schedule of what you eat in a day and show you, ‘Look, this is a sugar package you’ve consumed, and with diabetes you can’t handle eating this.’ I mean, more of these kinds of discussions, rather than just doing the clinical part: prick and check that your feet are clean and neat. [18]

Advice and measures from diabetes care, perceived as being overly governed by protocols, could conflict with individual circumstances. For example, one participant felt they were being referred to a dietitian simply because the DNs needed to check the box ‘has sent the patient to a dietitian.’

Shared experiences included being advised to stop eating at 6 PM, despite coming home from work around that time, or being encouraged to go to the gym though weightlifting was inappropriate due to specific health conditions. In contrast, participants expressed a desire for a more personalized, creative, and engaged approach. Furthermore, experiences of overly schematic diabetes care were perceived as manageable if enough time was allocated and a safe conversational environment established during the meetings with the healthcare professionals. One participant suggested that the DN and dietitian be allowed a freer, less rigid narrative, as the current evidence-based approach was seen as insufficient for providing meaningful advice and guidance on living with T2D.

#### Demands to take control of one’s own care

Based on a desire to maintain or improve well-being, participants actively shaped the prerequisites for better contact with diabetes care. In some cases, this was driven out of necessity and compulsion, such as when individual caregivers were perceived as neglecting needs and suggestions or lacking the appropriate competence or engagement. This had led to actions such as contacting the healthcare center manager with feedback, informing a new GP in writing about experiences and expectations, changing healthcare centre or GPs, or suggesting and negotiating specific medications or treatments:

There is nothing that the care has done that I haven’t come up with for them to do. [15]

In other cases, the demands to take control of one’s own care was driven by the desire to minimize contact with diabetes care, motivated by a strong wish to be independent and manage on their own. This was handled subtly, by leaving the visits quickly, not taking up more time than necessary with questions during a visit to diabetes care, or not seeking care unless absolutely necessary. The intention to minimize contact with diabetes care could be far-reaching. For example, one participant planned to cancel a visit for routine retinal screening, feeling the examination was unnecessary due to their perception of having good vision and believing that others might need the resources more.

#### Diabetes care is just a routine control

For some participants, contact with diabetes care meant no more than routine controls, and they expressed no specific expectations from a visit. While measurements and medical prescriptions were important to all participants, advice about lifestyle habits was considered as uninteresting or contrary to their own preferences. They were satisfied with their lives as they were and perceived their measurement values to be acceptable:

At first, I was given advice about lifestyle so that I should become aware. But then I probably do what I want anyway. [21]

One participant, who already had a healthy lifestyle and was at normal weight at diagnosis, found anything beyond checking values, clinical examination, and prescriptions to be redundant. They also felt that their contact with diabetes care was limited to these aspects. Viewing and expecting diabetes care as nothing more than routine check-ups was linked to having realistic expectations of life and living with T2D. These participants stated that they felt well, despite, in some cases, living with severe comorbidities.

## Discussion

The focus on experiences of contacts with diabetes care professionals among people living with T2D who are accessing diabetes care in the city of Malmö revealed that these experiences were oriented around relationships and structure. Participants expressed seeking room for their agency in their interactions with diabetes care professionals. Agency, defined as the belief in personal efficacy, is rooted in the core conviction that one has the power to effect change through one’s actions. It encompasses intentionality, forethought, self-reactiveness, and self-reflectiveness [35]. Lack of agency did not appear to be a problem in this study, but there was at times a lack of room to exercise it—pointing to a need for receptiveness among diabetes care professionals to patients’ voices and sufficient time during encounters. Sufficient time during regular encounters has been emphasized as one of the most important factors for perceived high-quality care when studied from the patients’ perspective [20]. Participants described a need to self-educate extensively due to a perceived lack of discussions with diabetes care professionals about their individual risk of developing complications or receiving none or over-simplified lifestyle guidance. This finding aligns with those from a Dutch study [36], where participants mainly felt they had to figure out everything about living with diabetes on their own. They perceived that healthcare professionals provided medical advice but did not explain how to manage T2D in daily life. Similarly, an English study involving persons living with early-onset T2D and healthcare professionals, described the need for extensive self-education. This was attributed to conflicting information, which in turn linked to fragmented healthcare and a lack of involvement from specialized care [37].

Experiences of shortcomings in discussions about lifestyle habits suggest healthcare professionals sometimes avoid this topic, possibly out of concern for implying guilt related to less healthy behaviours. However, such an approach may be overly cautious. A Danish interview study among individuals living with T2D described how doctors’ advice on losing weight contributed to a reassuring sense that someone was keeping an eye on them and that they had not been forgotten [38].

In a previous study on experiences of diabetes care, participants expressed they wanted to be listened to and that caregivers acknowledge their experiences, knowledge and desire for shared decision-making [18]. In our study, participants expressed a desire to be met with a more open or flexible narrative in the advice provided by diabetes care professionals. The wish to be met with a broader paradigm than the prevailing evidence-based one may reflect a need for enhanced shared decision-making in diabetes care encounters. Shared decision-making has been highlighted as fundamental in patient-centred T2D care, as evidence is necessary but never sufficient for making clinical decisions. This process also requires consideration of the person’s values, expectations, and context [39].

Previous research has shown positive psychosocial and beneficial biochemical outcomes from different group-based educations for persons living with T2D [40–42]. Typically, group-based education programs are delivered by a team of educators, a DN alone, or a dietitian alone, with the goals to improve self-efficacy and glycaemic control [41]. Examples of themes for the sessions are general issues concerning the disease, treatment, prevention of complications, blood glucose monitoring, diet, physical activity and daily foot care [22]. According to the Swedish National Board of Health and Welfare’s national guidelines for diabetes care, group-based education programs – supported by staff with subject expertise and pedagogical competence – should be offered for those affected by T2D [16]. However, only 24% of the primary healthcare units in Sweden offered such programs when last evaluated by the Swedish National Board of Health and Welfare [43]. The Swedish Association for Nurses in Diabetes Care has recently reported even lower figures (16%). In Skåne, the region where Malmö is situated, as few as 10% of the responding primary healthcare centres (23% of the contacted units) reported offering group-based education. Meanwhile, such education is offered to a greater extent by primary health care centers in e.g. the Stockholm region [44], which highlights differences in how diabetes care is provided in different parts and cities of the country. Among the reasons given for why group-based education was not offered at one’s unit were a lack of pedagogical competence [44], which points to an important area for development in the education of DNs.

In the current study, none of the participants reported being engaged in a group-based program for T2D within a healthcare setting, though a significant number expressed a desire to participate in such forums. The findings suggest that group-based education and support could fill an important gap in diabetes care. The absence of such initiatives may reflect limited resources within primary care. Involving municipalities in rehabilitation and support efforts could help bridge this gap, as has been done, for instance, in Denmark [28,45]. As group-based education and support appear to represent an unmet need in diabetes care in Malmö, the timely development and implementation of such initiatives are recommended, with consideration given to involving additional stakeholders in their delivery.

Measures aimed at achieving treatment goals according to guidelines and diabetes self-management education and support (DSMES) are cornerstones of diabetes care [16]. However, in the current study, diabetes care was sometimes perceived as overly protocol-driven, with insufficient consideration of individual factors and lifestyle. As a result, participants expressed a desire for more time during consultations—an observation that aligns with the findings of Edwall et al. [20], who emphasize that sufficient time during routine encounters is a key component of high-quality DN-led care. This aspect warrants attention in the present context.

T2D was perceived as a serious condition, and in light of this, a dualistic view of care levels emerged. Doctors in specialized care were seen as having deep knowledge and engagement. Not fitting the typical T2D profile led to reflections on whether a referral to an endocrinologist for a more individualized assessment might have been appropriate. This experience aligns with findings from the previously referenced study involving people with early-onset T2D and healthcare providers, where the need for specialist care was emphasized for persons who do not fit the typical T2D pattern, such as younger adults. It was identified that a GP care alone cannot meet the complex needs of people with early-onset T2D [37]. This finding may also be relevant in the Swedish context, based on patient experiences in the study.

Focusing on relational experiences, contact with diabetes care was more than just a momentary encounter: these meetings left lasting impressions. Feeling cared for as an individual was valued and seen as supportive in managing the disease, with healthcare professionals sometimes regarded as close allies. Previous studies have shown a consistent and significant positive relationship between interpersonal continuity of care and patient satisfaction [17,38,46], and long-lasting contact with a medical doctor appeared as a strong wish by people living with complications to T2D in a recent Belgian study [19]. Relational continuity has also been described by people living with T2D as a prerequisite for a good and open discussion between the patient and caregiver in diabetes care [18]. Subsequently, the lack of continuity in the relationship with the GP, as commonly found in the current study, calls for action.

Verbal maltreatment was experienced to a lesser extent, but it left a deep and lasting impact. Reflecting on being offensively confronted about obesity, even years later, brought feelings of sadness. When healthcare professionals comment negatively on diabetes management or related outcomes, this is experienced as demotivating for patients. This often contradicts the intention of supporting behavioural changes [47]. Using language that contributes to stigmatisation can make persons living with T2D less likely to seek follow-up care and more likely to experience psychological distress [48]. This needs to be critically reflected upon in every healthcare contact and interaction with persons with T2D.

## Strengths and limitations

Qualitative interviews were considered a well-suited method for exploring experiences of contacts with diabetes care professionals among persons living with T2D. Qualitative methods can provide rich and diverse data that explore *how* something works, offering insights that quantitative methods, which focus on *what* works, cannot provide [29]. By using an inductive approach, broad insights were gained that might not have emerged through a predefined theoretical framework [29]. Given the broad aim of the study and the use of convenience sampling, 21 persons were interviewed to ensure adequate information power [49]. To strengthen dependability, the same interviewer conducted all interviews, thereby facilitating consistency in data collection [32]. Additionally, a strength of this study lies in the inclusion of persons of various genders and ages from diverse backgrounds and with variations in the years since diagnosed. This diversity contributes to a rich variation of the phenomena under study, thereby strengthening the credibility of the findings [32]. The findings are presented together with quotations in the current study to provide a basis for the reader’s own interpretation and to enhance the credibility of the analysis. Furthermore, the analysis was conducted through a back and forth-discussion process within the research-team [32].

The interviewer (first author, JT), the second author (MAG), and the third author (EM) have backgrounds as RNs with experience in primary care. The fourth author (SZ), though experienced in diabetes research, did not have professional experience in clinical care, which may have mitigated the potential biased influence of pre-understanding on the analysis. The authors have extensive experience of conducting qualitative interview-studies. Further, MAG holds a PhD in diabetes care, while both EM and SZ are associated professors.

The findings can be considered for transferability to cities like Malmö, which are characterized by diverse populations and varying socioeconomic statuses, within a high-income country with mainly publicly funded healthcare [32].

Several limitations should be acknowledged. The inclusion of participants by DNs may constitute a risk for bias as the participants depend on their relationship with their caregiver. However, both positive and negative experiences were shared, thus indicating the participants felt safe to be honest in their accounts of their diabetes care contact experiences.

Further, semi-structured interviews provided in-depth data about experiences with the diabetes care. However, their use may have prompted the participants to share experiences they might not have disclosed in unstructured interviews. Although our analysis revealed experiences from participants across different socioeconomic groups, we were unable to capture the role of socioeconomic status in these varied experiences. This is an area we plan to investigate in a future study.

## Conclusion

Participants felt they were seeking room for individual needs in their interactions with diabetes care professionals. Diabetes care was in some cases considered protocol-driven which was experienced as not leaving enough room for more personal circumstances in the encounters. Contact with diabetes care professionals was perceived as supportive and made participants feel secure when there was a clear intention to adapt to individual needs, and when expectations and communication were clear.

Educational needs were, to some extent, perceived as unmet due to the lack of group-based formats and the sometimes overly simplified nature of the education provided. Efforts towards a systematic and broad implementation of group-based education and support programs for persons living with T2D in Malmö are recommended. A lack of continuity in contact with GPs was identified as a recurring concern which warrants further attention.
